# Supplementary material for: The Evonik-Mainz-Eye-Care-Study (EMECS): Design and Execution of the Screening Investigation
Source: PLoS One. 2014 Jun 10;9(6):e98538. doi: 10.1371/journal.pone.0098538 (PMC4051641; doi:10.1371/journal.pone.0098538)
Supplement: Table S1 — General medical history of all participants (n = 4183). (DOCX) [file pone.0098538.s001.docx]

**Table S1:** General medical history of all participants (n=4183).

|  | **40-44 years** | **45-49 years** | **50-54 years** | **55-59 years** | **≥60 Years** | **All together** |
| --- | --- | --- | --- | --- | --- | --- |
| **All together** | 1201 | 1287 | 1034 | 595 | 66 | 4183 |
| **Smoker** | | | | | | |
| No | 755 | 736 | 540 | 334 | 39 | 2404 |
| Yes | 224 | 267 | 225 | 104 | 7 | 827 |
| Former smoker | 218 | 278 | 261 | 154 | 19 | 930 |
| Missing data | 4 | 6 | 8 | 3 | 1 | 22 |
| **Tobacco products** | | | | | | |
| Cigarette | 413 | 505 | 443 | 238 | 20 | 1619 |
| Others | 12 | 24 | 25 | 12 | 3 | 76 |
| Missing data | 17 | 16 | 18 | 8 | 3 | 62 |
| **Pack years of cigarette smokers and former cigarette smokers together** | | | | | | |
| Mean | 16.3 | 20.6 | 24.2 | 23.7 | 31.6 | 21.1 |
| **Treatment of blood hypertension** | | | | | | |
| Yes | 93 | 198 | 275 | 195 | 21 | 782 |
| No | 1106 | 1087 | 756 | 400 | 45 | 3394 |
| Missing Data | 2 | 2 | 3 | 0 | 0 | 7 |
| **Treatment with Cortison** | | | | | | |
| No | 1165 | 1242 | 999 | 573 | 66 | 4045 |
| With tablets | 5 | 4 | 4 | 4 | 0 | 17 |
| With aerosol | 16 | 30 | 17 | 11 | 0 | 74 |
| With injection | 5 | 2 | 4 | 0 | 0 | 11 |
| Missing data | 10 | 9 | 10 | 7 | 0 | 36 |
| **Diabetes mellitus** | | | | | | |
| No | 1177 | 1250 | 984 | 555 | 62 | 4028 |
| ≤ 5 years | 5 | 14 | 25 | 11 | 3 | 58 |
| > 5 years | 5 | 13 | 12 | 21 | 0 | 51 |
| If yes, therapy with insulin | 3 | 11 | 5 | 8 | 0 | 27 |
| Missing data | 14 | 10 | 13 | 8 | 1 | 46 |
| **Hypercholesterolemia** | | | | | | |
| Yes | 264 | 323 | 342 | 363 | 19 | 1311 |
| No | 895 | 924 | 650 | 220 | 47 | 2736 |
| Missing data | 42 | 40 | 42 | 12 | 0 | 136 |
| **Cardiac disease** | | | | | | |
| Yes | 12 | 33 | 33 | 31 | 4 | 113 |
| No | 1189 | 1254 | 1001 | 564 | 62 | 4070 |
| **Pulmonal disease** | | | | | | |
| Yes | 55 | 57 | 46 | 31 | 1 | 190 |
| No | 1146 | 1230 | 988 | 564 | 65 | 3993 |
| **Hematological disease** | | | | | | |
| Yes | 5 | 2 | 4 | 3 | 2 | 16 |
| No | 1196 | 1285 | 1030 | 592 | 64 | 4167 |
| **Renal disease** | | | | | | |
| Yes | 7 | 3 | 3 | 5 | 1 | 19 |
| No | 1194 | 1284 | 1031 | 590 | 65 | 4164 |
| **Metabolic disease** | | | | | | |
| Yes | 115 | 135 | 130 | 86 | 15 | 481 |
| No | 1086 | 1152 | 904 | 509 | 51 | 3702 |
| **Rheumatological disease** | | | | | | |
| Yes | 13 | 16 | 17 | 12 | 0 | 58 |
| No | 1188 | 1271 | 1017 | 583 | 66 | 4125 |
| **Neurological disease** | | | | | | |
| Yes | 6 | 3 | 11 | 8 | 2 | 30 |
| No | 1195 | 1284 | 1023 | 587 | 64 | 4153 |
| **Psychiatric disease** | | | | | | |
| Yes | 9 | 17 | 11 | 9 | 1 | 47 |
| No | 1192 | 1270 | 1023 | 586 | 65 | 4136 |
| **Allergies** | | | | | | |
| Yes | 564 | 535 | 381 | 213 | 20 | 1713 |
| No | 598 | 722 | 622 | 376 | 46 | 2364 |
| Missing data | 4 | 30 | 31 | 6 | 0 | 106 |
| **If yes, aigainst drugs*** |  |  |  |  |  |  |
| Yes | 85 | 102 | 79 | 36 | 4 | 306 |
| No | 1116 | 1185 | 955 | 559 | 62 | 3877 |
| **If yes, against food*** |  |  |  |  |  |  |
| Yes | 92 | 90 | 55 | 43 | 2 | 282 |
| No | 1109 | 1197 | 979 | 552 | 64 | 3901 |
| **If yes, against pollen*** |  |  |  |  |  |  |
| Yes | 354 | 306 | 206 | 109 | 12 | 978 |
| No | 856 | 981 | 828 | 486 | 54 | 3205 |
| **If yes, against material contact*** |  |  |  |  |  |  |
| Yes | 89 | 71 | 42 | 26 | 2 | 230 |
| No | 1112 | 1216 | 992 | 569 | 64 | 3953 |
| **If yes, against others*** |  |  |  |  |  |  |
| Yes | 184 | 174 | 118 | 70 | 5 | 551 |
| No | 1017 | 1113 | 916 | 525 | 61 | 3632 |

* Multiple answers possible
