# Supplementary material for: The Evonik-Mainz-Eye-Care-Study (EMECS): Design and Execution of the Screening Investigation
Source: PLoS One. 2014 Jun 10;9(6):e98538. doi: 10.1371/journal.pone.0098538 (PMC4051641; doi:10.1371/journal.pone.0098538)
Supplement: Table S2 — General examination of all participants (n = 4183). (DOCX) [file pone.0098538.s002.docx]

**Table S2:** General examination of all participants (n=4183).

|  | **40-44 years** | **45-49 years** | **50-54 years** | **55-59 years** | **≥60**  **Years** | **All together** |
| --- | --- | --- | --- | --- | --- | --- |
| **All together** | 1201 | 1287 | 1034 | 595 | 66 | 4183 |
| **Body height** | | | | | | |
| Mean [cm] | 176.9 | 177.3 | 176.3 | 174.6 | 175.1 | 176.5 |
| Missing data | 2 | 1 | 4 | 1 | 0 | 8 |
| **Body weight** | | | | | | |
| Mean [kg] | 82.1 | 83.8 | 84.6 | 83.8 | 84.4 | 83.5 |
| Missing data | 2 | 1 | 4 | 1 | 0 | 8 |
| **Body mass index** | | | | | | |
| Mean | 26.1 | 26.6 | 27.1 | 27.4 | 27.5 | 26.7 |
| **Waist circumference** | | | | | | |
| Mean [cm] | 93.8 | 96.4 | 98.3 | 98.9 | 99.0 | 96.5 |
| Missing data | 3 | 0 | 2 | 2 | 0 | 7 |
| **RR systolic** | | | | | | |
| Mean [mmHg] | 129.1 | 132.5 | 135.2 | 137.5 | 141.2 | 133.1 |
| Missing data | 4 | 1 | 1 | 1 | 0 | 7 |
| **RR diastolic** | | | | | | |
| Mean [mmHg] | 81.2 | 82.7 | 83.9 | 84.0 | 84.9 | 82.8 |
| Missing data | 5 | 2 | 2 | 3 | 0 | 12 |
| **Mean arterial pressure** | | | | | | |
| Mean [mmHg] | 97.2 | 99.3 | 101.0 | 101.8 | 103.6 | 99.5 |
| Missing data | 5 | 2 | 2 | 3 | 0 | 12 |
