# Supplementary material for: The Evonik-Mainz-Eye-Care-Study (EMECS): Design and Execution of the Screening Investigation
Source: PLoS One. 2014 Jun 10;9(6):e98538. doi: 10.1371/journal.pone.0098538 (PMC4051641; doi:10.1371/journal.pone.0098538)
Supplement: Table S3 — Ophthalmological history of all participants (n = 4183). (DOCX) [file pone.0098538.s003.docx]

**Table S3:** Ophthalmological history of all participants (n=4183).

|  | **40-44 years** | **45-49 years** | **50-54 years** | **55-59 years** | **≥60**  **Years** | **All together** |
| --- | --- | --- | --- | --- | --- | --- |
| **All together** | 1201 | 1287 | 1034 | 595 | 66 | 4183 |
| **History of glaucoma** | | | | | | |
| No | 1194 | 1273 | 1018 | 586 | 63 | 4134 |
| Yes, medicinal treatment to reduce IOP | 4 | 6 | 11 | 2 | 3 | 26 |
| Yes, glaucoma surgery | 1 | 1 | 1 | 0 | 0 | 3 |
| Yes, treatment and surgery | 0 | 1 | 0 | 0 | 0 | 1 |
| Yes, glaucoma but no therapy | 1 | 2 | 1 | 2 | 0 | 6 |
| Yes,ocular hypertension without treatment | 1 | 4 | 3 | 5 | 0 | 13 |
| **Glaucoma in relation in the first degree** | | | | | | |
| Only parents | 84 | 83 | 62 | 40 | 2 | 271 |
| Only siblings | 7 | 4 | 5 | 2 | 0 | 18 |
| Parents and siblings | 2 | 4 | 3 | 3 | 0 | 12 |
| No relation | 1091 | 1177 | 941 | 534 | 63 | 3806 |
| Missing data | 17 | 19 | 23 | 16 | 1 | 76 |
| **Known eye disease*** | | | | | | |
| Diabetic Retinopathy | 0 | 2 | 1 | 2 | 0 | 5 |
| Age-Related Macular Degeneration | 0 | 0 | 2 | 1 | 0 | 3 |
| Amblyopia | 8 | 8 | 7 | 6 | 0 | 29 |
| Cataract | 4 | 2 | 3 | 0 | 1 | 10 |
| Condition after corneal injury | 28 | 35 | 26 | 17 | 2 | 108 |
| Uveitis in history | 8 | 8 | 3 | 1 | 0 | 20 |
| Recurrent conjunctivitis | 3 | 4 | 2 | 3 | 2 | 14 |
| Peripheral retinal degeneration | 18 | 28 | 29 | 19 | 0 | 94 |
| Retinal angiopathy | 1 | 2 | 2 | 3 | 1 | 9 |
| Pseudophakia | 1 | 7 | 6 | 10 | 2 | 26 |
| Condition after contusio bulbi | 4 | 2 | 4 | 4 | 0 | 14 |
| Endocrine orbitopathy | 1 | 0 | 1 | 0 | 0 | 2 |
| Pterygium | 0 | 1 | 4 | 0 | 1 | 6 |
| Condition after neuritis nervi optici | 3 | 0 | 0 | 2 | 0 | 5 |
| Condition after chemical burn | 1 | 4 | 2 | 1 | 2 | 10 |
| Others | 24 | 24 | 19 | 20 | 0 | 87 |
| **History of eye surgery*** | | | | | | |
| LASIK | 11 | 7 | 5 | 2 | 0 | 25 |
| Cataract surgery | 2 | 7 | 6 | 10 | 2 | 27 |
| Corneal foreign bodies | 22 | 31 | 21 | 12 | 1 | 87 |
| Retinal laser coagulation | 13 | 28 | 22 | 19 | 0 | 82 |
| Lid surgery | 2 | 1 | 3 | 1 | 0 | 7 |
| Retinal surgery | 2 | 0 | 4 | 0 | 0 | 6 |
| Others | 2 | 3 | 8 | 4 | 0 | 17 |
| **Eye drops** | | | | | | |
| None /missing | 1173 | 1251 | 997 | 571 | 59 | 4051 |
| Artificial tears | 19 | 22 | 20 | 17 | 2 | 80 |
| Anti-allergic | 3 | 2 | 2 | 1 | 1 | 9 |
| Anti-inflammatoric | 1 | 1 | 2 | 1 | 1 | 6 |
| Anti-glaucomatous | 4 | 7 | 11 | 2 | 3 | 27 |
| With topical steroids | 0 | 1 | 0 | 1 | 0 | 2 |
| Others | 1 | 3 | 2 | 2 | 0 | 8 |
| **Strabismus right and /or left** | | | | | | |
| No | 1099 | 1188 | 976 | 560 | 61 | 3884 |
| Yes | 47 | 55 | 24 | 18 | 2 | 146 |
| Strabismus surgery before | 22 | 9 | 11 | 5 | 2 | 49 |
| Missing data | 33 | 35 | 23 | 12 | 1 | 104 |
| **Visual aid** | | | | | | |
| No | 489 | 285 | 39 | 7 | 1 | 821 |
| Reading spectacles | 90 | 300 | 356 | 168 | 15 | 929 |
| Distance spectacles | 468 | 313 | 123 | 49 | 5 | 958 |
| Distance and reading spectacles | 144 | 382 | 515 | 369 | 45 | 1455 |
| Spectacles, unspecified | 2 | 3 | 0 | 0 | 0 | 5 |
| Missing Data | 8 | 4 | 1 | 2 | 0 | 15 |
| **Last visit to ophthalmologist** | | | | | | |
| ≤ 3 Years | 607 | 768 | 690 | 403 | 45 | 2513 |
| > 3 Years | 580 | 506 | 334 | 188 | 21 | 1629 |
| Missing data | 14 | 13 | 10 | 4 | 0 | 41 |

* Several answers per participant were possible. Answers were categorized by ophthalmological expert opinion (LB).
