# Supplementary material for: Correction: The Evonik-Mainz-Eye-Care-Study (EMECS): Design and Execution of the Screening Investigation
Source: PLoS One. 2014 Aug 25;9(8):e107083. doi: 10.1371/journal.pone.0107083 (PMC4143346; doi:10.1371/journal.pone.0107083)
Supplement: File S2 — Republished, corrected article.(PDF) [file pone.0107083.s002.pdf]

PLOS | ONE

The Evonik-Mainz-Eye-Care-Study (EMECS): Design and Execution of the Screening Investigation

Lorenz Barleon[1]*, Jochen Wahl[1], Peter Morfeld[2,3], Claudia Deters[1], Andrea Lichtmeß[1], Sibylle Haas-Brähler[4], Uta Müller[5], Rolf Breitstadt[5], Norbert Pfeiffer[1]

1 Department of Ophthalmology, University Medical Center, Johannes-Gutenberg-University, Mainz, Germany, **2** Institute for Occupational Medicine, Environmental Medicine and Prevention Research, University of Cologne, Köln, Germany, **3** Institute for Occupational Epidemiology and Risk Assessment of Evonik Industries, Essen, Germany, **4** Occupational Health, Evonik Industries, Hanau, Germany, **5** Occupational Health, Evonik Industries, Essen, Germany

Abstract

Purpose: To determine if screening for major ophthalmological diseases is feasible within the frame of routine occupational medicine examinations in a large working population.

Methods: 13037 employees of Evonik Industries aged 40 to 65 years were invited to be screened for major ophthalmological diseases (glaucoma, age related macular degeneration and diabetic retinopathy between June 2007 and March 2008 within an extended setting of occupational medicine. Ophthalmological examinations consisted of visual acuity, objective refraction, pachymetry, tonometry, perimetry (frequency doubling technology), confocal scanning laser ophthalmoscopy and digital fundus photography. Participants responded to a questionnaire addressing history of ocular and general diseases and social history.

Results: 4183 participants (961 female and 3222 male) were examined at 13 different sites. Response rates for eligible persons at those sites ranged from 17.9 to 60.5% but were in part limited by availability of examination slots. Average age of participants was 48.4±5.4 years (mean ± SD). 4147 out of 4183 subjects (99.1%) had a visual acuity ≥0.5 in the better eye and 3665 out of 4183 (87.6%) subjects had a visual acuity ≥0.8 in the better eye. 1629 participants (38.9%) had previously not been seen by an ophthalmologist at all or not within the last three years.

Conclusion: This article describes the study design and basic characteristics of study participants within a large occupational medicine based screening study for ophthalmological diseases. Response rates exceeded expectations and were limiting examination capacity. Meaningful data could be obtained for almost all participants. We reached participants who previously had not received ophthalmic care. Thus, ophthalmological screening appears to be feasible within the frame of routine occupational medicine examinations.

Citation: Barleon L, Wahl J, Morfeld P, Deters C, Lichtmeß A, et al. (2014) The Evonik-Mainz-Eye-Care-Study (EMECS): Design and Execution of the Screening Investigation. PLoS ONE 9(6): e98538. doi:10.1371/journal.pone.0098538

Editor: Thomas Behrens, Universität Bochum, Germany

Received November 5, 2013; **Accepted** May 5, 2014; **Published** June 10, 2014

Copyright: © 2014 Barleon et al. This is an open-access article distributed under the terms of the Creative Commons Attribution License, which permits unrestricted use, distribution, and reproduction in any medium, provided the original author and source are credited.

Funding: This investigation was sponsored by a grant from Evonik Industries. The funders had no role in study design, data collection and analysis, decision to publish, or preparation of the manuscript.

Competing Interests: One or more authors have an affiliation to the commercial funders of this research study (Evonik Industries). The corresponding author declares, on behalf of all authors, that there are no competing interests. Evonik Industries provided an unrestricted grant for this study. Evonik Industries provided some of the equipment, qualified personnel, transportation and accommodation. The interest of Evonik Industries was to supply preventive health care for their employees. Evonik Industries have no business activity in ophthalmology. Apart from the co-authors, the funders had no role in study design, data collection and analysis, decision to publish, or preparation of the manuscript. There is no conflict of interest of the sponsor or the members of Evonik Industries. This does not alter the authors' adherence to PLOS ONE policies on sharing data and materials.

* E-mail: L.Barleon@diak-ka.de

Introduction

Major causes of loss of visual function and even blindness in most countries worldwide are glaucoma, age-related macular degeneration (ARMD) and diabetic retinopathy (DRP) [1–3]. Often, much of visual function is lost before the diseases are even diagnosed. Furthermore, the number of patients, who suffer from these major ophthalmological diseases, will increase in an aging population of industrialised countries [4–7].

Also, the workforce will continually age [8,9] and requirements for an optimal visual capacity of the workers, when working with modern software products [10], will increase. In the working age group, diabetic retinopathy is a leading cause of visual impairment [11]. Hence, major causes of loss of visual function may even become a relevant problem for the workforce and will challenge occupational medicine.

Thus, screening for such eye diseases has been suggested. However, studies indicated that screening of the general population, especially for glaucoma [12–15] would be rather expensive and relatively inefficient. In case of ARMD even accurate and standardised screening methods have not been established [16].

Therefore, it was suggested that screening should be directed at groups at risk [13,17]. Apart from DRP [7], procedures for identifying those at risk are few [13].

One option to make screening for eye diseases more cost efficient might be to combine screening with other routine medical examinations [18].

On the basis of legal requirements, companies in Germany offer occupational health supervision and medical exams to their employees to detect functional disorders that may influence the employee's ability to work in his/her specific job.

These include the so called G-examinations, G-37 for visual display unit (VDU) workplaces (to performed at least once every 3 years for ages >40 years) and G-25 for driving, controlling and monitoring work (repeated every 2 to 3 years for ages >40 years and every 1 to 2 years for ages >60 years).

These G examinations include near and distance visual acuity (G-25, G-37), visual field (automated suprathreshold perimetry) (G-25, G-37), examination of stereopsis (G-25, G-37), color vision (G-25, G-37) and a mesopic vision and glare test (G-25) [19] (Table 1). Similar procedures are recommended in other countries, for example in the United Kingdom by the Health and Safety Executive [20,21] and in the USA by the Occupational Safety and Health Administration [22,23]. In addition there are specific visual requirements for other occupational groups in the USA (e.g. commercial truck drivers [24] or pilots [25]).

In 2002, 40% of all occupational examinations in Germany, which corresponds to more than 2 million occupational examinations, were G-25 (15%) and G-37 (25%) examinations [26].

In view of these large numbers of occupational health examinations for a large proportion of the workforce we asked if these examinations might be augmented to render a feasible screening for major eye diseases realizable within the frame of routine occupational medicine examinations.

Therefore, we designed the Evonik-Mainz-Eye-Care-Study (EMECS) and augmented the occupational medicine examination by ophthalmological procedures such as measuring intraocular pressure, pachymetry, confocal scanning laser ophthalmoscopy and fundus photography.

Apart from a pilot study with 392 participants from Deutsche Lufthansa AG [27] EMECS is the first large study with such a screening concept using modern ophthalmological examination techniques within the infrastructure of the existing occupational health care system. However, similar approaches, like screening for glaucoma or driving ability among workers, have been reported [28,29].

This article describes the study protocol, design, how the screening was executed and baseline characteristics of the cohort.

Designs and Methods

The Evonik-Mainz-Eye-Care-Study (EMECS) was performed in cooperation with the Department of Occupational Health of Evonik Industries, which is one of the largest companies in the chemical sector in Germany, and the Department of Ophthalmology at Mainz University Medical Centers.

The study was designed as a multi-site, single-center, cross-sectional study.

The examination was conducted as a one-time screening and consisted of visual acuity, objective refraction, pachymetry, tonometry, perimetry (frequency doubling technology), confocal scanning laser ophthalmoscopy and digital fundus photography.

Recruitment

Prior to all examinations identifier, age and gender of all employees at 13 out of 16 different German sites of the company were listed (overview in Table 2). Sites were situated in the Southern and Western parts of Germany. All employees between the ages of 40 and 65 years were invited to be examined. Apart from age, no other exclusion criterion was defined. The employees were informed via an advertising campaign that included intranet information, posters at highly frequented spots and information by e-mail. The campaign used glaucoma topics as an "eye catcher". Within a timeframe of 10 months, and based on an expected participation of 30%, we aimed to examine roughly 4000 of all invited employees (13037). For this study we did not plan a complete survey of the whole workforce.

Medical Examination Procedure

For all examinations, including the relocation of all equipment and examination units from site to site, a time period of 10 months (June 2007 – March 2008) was planned. The study was proceeded by a pilot phase including 50 test individuals, which were recruited at site 1 (first site investigated, see Table 2).

All participants were examined by one ophthalmologist (LB) and two assistants (CD, AL), all members of the Department of Ophthalmology at Mainz University Medical Center. All exam-

Table 1. Ophthalmological examinations within occupational health supervision in Germany.

Examinations	G-25*	G-37**
**Regarding employees**	driving, controlling and monitoring work	visual display unit workplaces
**Interval of Examination**	every 2–3 years for age >40 years and every 1–2 years for age >60 years	at least every 3 years for age >40 years
**near and distance visual acuity**	included	included
**automated supratreshold perimetry**	included	included
**stereopsis**	included	included
**color vision**	included	included
**mesopic vision and glare test**	included	**not** included

*G-25 for driving, controlling and monitoring work and **G-37 for visual display unit (VDU) workplaces.
doi:10.1371/journal.pone.0098538.t001

Table 2. Participation rate for each site and in total.

Sites of Evonik Industries	Number of employees with age >40 years	Available examination time at each site (in weeks)	Demand for examination greater than available resources for examination	Number of study participants	Participation rate (in %)
**Site 1[1]**	662	3	No	247	37.3
**Site 2[2]**	1336	5,5	Yes	641	48.0
**Site 3[2]**	409	2	No	230	56.2
**Site 4[2]**	342	2	No	207	60.5
**Site 5[2]**	823	3,5	No	374	45.4
**Site 6[2]**	3795	8	Yes	877	23.1
**Site 7[2]**	703	2	No	266	37.8
**Site 8[1]**	1187	2	No	213	17.9
**Site 9[2]**	453	1,5	No	203	44.8
**Site 10[2]**	864	2,5	No	293	33.9
**Site 11[2]**	728	2	No	296	40.7
**Site 12[2]**	1149	2	Yes	228	19.8
**Site 13[2]**	584	1	Yes	108	18.5
**Total**	13037	38,5	–	4183	32,1

[1]administration site,
[2]production site.
doi:10.1371/journal.pone.0098538.t002

inations included medical history, general examinations and ophthalmologic examinations.

Medical History

Every participant underwent a standardised interview to collect personal data (age, gender, skin color) and to fill out a medical questionnaire. The medical questionnaire covered the ophthalmological and the general medical history. All interviews were performed by a trained member of each local Department of Occupational Health of Evonik Industries.

The general medical history consisted of questions concerning:

- allergies
- smoking habits
- history of chronic disease (including diabetes mellitus, systemic hypertension, hypercholesterolemia)
- use of medications, specifically antihypertensive drugs, antidiabetic medications and steroids.

The ophthalmological history consisted of questions concerning:

- previous or present eye diseases, with a special focus on glaucoma history
- family history of glaucoma
- history of eye surgery
- application of eye drops
- use of visual aids (glasses for distance and/or reading)
- previous visit to an ophthalmologist ($>$ or $\leq$3 years).

Furthermore, a questionnaire including 15 questions/items about software ergonomics and self-reported health problems was filled out by every participant.

General Examination

The general medical examination consisted of measurements of body weight, body height, waist circumference and blood pressure (systolic and diastolic in sitting position). For the indirect blood measurement (non-invasive blood pressure, NIPD) we used an automatic hemodynamometer (boso-carat professional, Jungingen, Germany).

For measuring the waist circumference we used a flexible measuring tape (unit in cm). All general examinations were performed by a member of the Department of Occupational Health of Evonik Industries prior to all ophthalmological examinations.

Ophthalmologic Examination

Requirement for all examination was a non-contact method and a performance in miosis to avoid any disablement for work. Furthermore the selection of ophthalmological examination tools followed the demand, that they also can be handled solitary by assistance personnel for screening examinations in the future. In particular we did not include a screening of cataract. Because we referred workers to an ophthalmologist if visual acuity was less than 0.8. We believed that all cases of cataract will be identified and handled although we did not screen for cataract.

The examinations, in the order of performance, consisted of determination of visual acuity, objective refraction, central corneal thickness (CCT), intraocular pressure (IOP), and visual field for both eyes. For diagnostic imaging, confocal scanning laser ophthalmoscopy and digital fundus photography were performed for both eyes. All examinations started with the right eye. Examinations were performed during the whole working day and the time of examination differed between individuals.

Visual Acuity and Objective Refraction

Visual acuity of each participant was determined by using her/his own visual aid (glasses, contact lenses). The monocular visual acuity for distance for both eyes using Landolt rings was tested using an Oculus Binoptometer III (Wetzlar, Germany). Objective refraction was obtained using a hand-held auto refractometer (Retinomax 2, Nikon Corp., Japan).

Requirement of visual acuity for distance for G-25 at its highest performance level is at least 0.7 for one eye and at least 0.5 for the other eye or at least binocular 0.8 or at least 0.7 in case of monocular vision after individual case assessment.

According to G-37 the minimum requirement for visual acuity for both, distance and near vision, is defined as follows: at least 0.8 for one eye and at least 0.8 for the other eye and at least 0.8 binocular [19].

Requirements of visual acuity to achieve driving ability (e.g. motor car, class A, B, M, L, S, T) by the German Ophtalmological Society [30] is 0.5 of the better eye or binocular 0.5, accordingly to the European Community [31].

To simplify, regarding the above mentioned facts, we used two classifications of visual acuity for distance for further analysis:

1. visual acuity $\geq$0.5 for the better eye
2. visual acuity $\geq$0.8 for the better eye.

But a recommendation to consult a general ophthalmologist was already made, if a participant had a visual acuity $<$0.8 in one eye.

Pachymetry

Central corneal thickness (CCT) for both eyes was determined by optical coherence pachymetry (OCP, 4-Optics, Heidelberg Engineering GmbH, Heidelberg, Germany).

Tonometry

Intraocular pressure (IOP) was measured using a non-contact tonometer (AT 555, Reichert Ophthalmic Instruments, Depew, NY, USA). The intraocular pressure was measured 3 times and the mean of these 3 measurements was used for further analysis. Each participant was categorised as ocular hypertensive, if the mean IOP was higher than 21 mmHg in at least one eye.

Perimetry

For perimetric examination we used frequency doubling technology (FDT) (Humphrey FDT, Carl Zeiss Meditec, Jena Germany) for both eyes. We used the program C-20-5 with 17 screen patterns. For classification we used the estimated probability value P (P$\geq$5%: within normal limits; P$<$5%: mild relative loss; P$<$2%: moderate relative loss, P$<$1%: severe loss). We defined separately for each eye, that a FDT C20-5 result was unreliable if fixation errors (FE) or false positive errors (FPE) were greater than 1 out of 3 catch trials ($>$1/3).

Confocal Scanning Laser Ophthalmoscopy

For confocal scanning laser ophthalmoscopy we used the Heidelberg Retina Tomograph 3.0 (HRT 3.0, Heidelberg Engineering, Heidelberg, Germany) in both eyes. To obtain sufficient quality of HRT images a standard deviation of every pixel of topography smaller than 30 μm was chosen according to manufacturer's guidelines. In case of a standard deviation greater than 30 μm, the measurement was repeated once. If the second measurement had a standard deviation greater than 30 μm, the affected eye was excluded from HRT analysis.

Fundus Photography

A 45° fundus photography was performed using a non-mydriatic retinal camera (Non-Mydriatic Retinal Camera CR-DGi, Canon Inc., Japan). The room was shaded and the picture was repeated, if necessary.

Reading Center

All results, collected per day, were sent to the Department of Ophthalmology at Mainz University Medical Center, where an evaluation was performed promptly by an experienced ophthalmologist (JW). Diseases of main interest were glaucoma, age-related macular degeneration (ARMD) and diabetic retinopathy (DRP).

Both eyes were evaluated. The overall evaluation was positive if at least one eye was categorised as positive.

Evaluation for Glaucoma

Glaucoma suspects were identified on the basis of the evaluation of optic disc photography, IOP and FDT. The optic disc was categorised by size, cup-disc-ratio (CDR), ISNT-rule, morphology of excavation, disc hemorrhages and asymmetry between eyes. The ISNT rule in non-glaucomatous eyes shows a characteristic configuration for nerve fiber layer thickness (Inferior $\geq$ Superior $\geq$ Nasal $\geq$ Temporal).

All subjects were categorised in "non-glaucoma suspects", "glaucoma suspects" (differentiating between possible or probable glaucoma cases) or no grading because of missing data.

All participants with glaucoma suspects were recommended to consult a general ophthalmologist.

Evaluation for Age-Related Macular Degeneration (ARMD)

ARMD suspects were identified by evaluation of the 45° fundus photography.

Grading of ARMD changes based on the presence or absence of ARMD signs within a radius of two discs diameter from the fovea and were clinically graded, as illustrated in table 3. Age-related changes in the macular region were categorised as early and late ARMD. Early ARMD included stages 1a, 1b, 2a, 2b and 3. Late ARMD comprises neovascular (haemorrhagic, serous, fibrous) and atrophic changes (stages 4a and 4b). Stage 5, maculopathy not related to ARMD, included lesions that were considered to be the result of generalized disease, such as vascular diseases (retinal vein occlusions), diabetic maculopathy, myopic degeneration, trauma, macular pucker or chorioretinitis. These lesions were excluded from ARMD grading. Small hard drusen were not included in the ARMD diagnosis.

All participants from stage 1 and higher were recommended to consult a general ophthalmologist.

Evaluation for Diabetic Retinopathy (DRP)

DRP suspects were identified by evaluation of the 45° fundus photography. The central retina was evaluated for the existence of microaneurysms, intraretinal microvascular abnormalities (IRMA), venous beading, retinal hemorrhages, soft or hard exudates, new vessels, fibrous proliferations, macular edema. Based on ETDRS grading [32] all subjects were categorised in non diabetic retinopathy, mild non-proliferative diabetic retinopathy, moderate non-proliferative retinopathy, severe non-proliferative retinopathy, proliferative retinopathy and non diabetic maculopathy, diabetic maculopathy or no grading because of missing data. We were not able to categorise macular edema by using fundusphotography.

All participants with any fundus changing due diabetes were recommended to consult a general ophthalmologist.

Evaluation for other Diseases

Additional noticeable findings such as naevi or fundus hypertonicus were also documented.

Table 3. Classification of stages of age-related macula degeneration (ARMD).

Stage	Legend
**0a**	no signs of ARMD at all
**0b**	hard drusen (<63 µm, ≤10) only
**0c**	hard drusen (<63 µm, >10) only
**1a**	soft distinct drusen (≥63 µm) only
**1b**	pigmentary abnormalities only, no soft drusen (≥63 µm)
**2a**	soft indistinct drusen (≥125 µm) or reticular drusen only
**2b**	soft distinct drusen (≥63 µm) with pigmentary abnormalities
**3**	soft indistinct (≥125 µm) or reticular drusen with pigmentary abnormalities
**4a**	atrophic ARMD
**4b**	neovascular ARMD
**5**	maculopathy unrelated to ARMD
**6a**	cannot grade (photo quality)
**6b**	cannot grade (obscuring lesion)
**6c**	cannot grade (missing photo)

doi:10.1371/journal.pone.0098538.t003

After the screening exam and evaluation every participant received a letter reporting results and noteworthy findings. In case of abnormalities in fundus photography, perimetry and/or intraocular pressure, as well as low visual acuity <0.8, a recommendation was given to consult an ophthalmologist for further investigation (Table 4).

Data Protection Issues

All individual data of each participant were encrypted with a barcode at each local Department of Occupational Health of Evonik Industries (pseudonomization). Immediately after the medical examination all recorded data, collected under each related barcode, were sent to the Department of Ophthalmology at Mainz University for evaluation. Afterwards the results and recommendations were sent back to the corresponding local Department of Occupational Health of Evonik Industries, where a re-identification of the personal data was performed. Pseudonominised individual data were transferred to the Institute for Occupational Epidemiology and Risk Assessment of Evonik Industries for analyses.

Ethics Statements

All participants gave written informed consent before entry into the study.

No open personal data were available at the University of Mainz and the additional medical examination was covered by the regulations of occupational medicine. All examinations were non-invasive.

The study protocol and data protection procedures were submitted to and accepted by the data protection office of DEGUSSA (K. Gowig, Head of Department of Data Protection, RAG-Beteiligungs-AG, Essen Germany). At that time this was the responsible institutional review board. This review board approved the study protocol. In the meantime, DEGUSSA became part of Evonik Industries. No new ethics committee approval was obtained or deemed necessary after this change.

Table 4. Ophthalmological history subdivided in glaucoma, ocular hypertension, diabetic retinopathy, age-related macular degeneration together with the last visit to ophthalmologist (n = 4183).

	**40–44 years**	**45–49 years**	**50–54 years**	**55–59 years**	**≥60 Years**	**All together**
**All together**	1201	1287	1034	595	66	4183
**Opthalmological history**						
Glaucoma	6	10	13	4	3	36
Ocular Hypertension	1	4	3	5	0	13
Diabetic Retinopathy	0	2	1	2	0	5
Age-Related Macular Degeneration	0	0	2	1	0	3
**Last visit to ophthalmologist**						
≤3 Years	607	768	690	403	45	2513
>3 Years	580	506	334	188	21	1629
Missing data	14	13	10	4	0	41

doi:10.1371/journal.pone.0098538.t004

All study procedures adhered to the recommendations of the Declaration of Helsinki.

Statistical Methods

All data underwent a quality control by the Institute for Occupational Epidemiology and Risk Assessment of Evonik Industries and were checked for completeness and correctness by plausibility controls. Descriptive statistics were calculated including means and standard deviations (mean ± standard deviation) and empirical distributions across categories of variables. Fisher's exact test was used in 2×2 table comparisons [33]. Statistical analyses were performed using Stata 11 [34]. A significance level of 5% was chosen.

Results

Participation Rates and Demographic Description

13037 out of 33258 employees of the company were 40 years or older (40–49 years: 59.1%, 50–59 years: 39.2%, 60–65 years: 1.7%). 16.2% out of these 13037 were female and 83.8% were male.

In this study we were able to examine 4183 (32.1%) out of these 13037 employees.

The participation rate of the 13 sites varied from 17.9% to 60.5%. At site no. 2, 6, 12 and 13 the demand for examination was greater than the available slots for examinations (Table 2). At these sites all employees were included on a "first come, first served" basis.

961 (23.0%) were female and 3222 (77.0%) were male participants (Table 5). 3723 employees worked at production sites and 460 did administrative work. The percentage of female employees was higher at sites with administrative function (36.5%) than at production sites (21.3%) (Table 6). Mean age of all participants was 48.4 years±5.4 years (from 40 to 65 years), 98.4% of all participants were in the age-group from 40 to 59 years. 98.9% of all participants were Caucasian (Table 5).

Detailed results of general medical history and general examinations are published in Tables S1 and S2.

Ophthalmological History

36 out of 4183 participants (0.9%) gave a history of glaucoma and 13 out of 4183 participants (0.3%) had known ocular hypertension. Five participants (0.1%) gave a history of having diabetic retinopathy. Three participants (0.1%) reported having age-related macular degeneration.

38.9% (n = 1629) of all participants had not been seen by an ophthalmologist for more than 3 years or never before (Table 4). This was different for women (31.7% of all participating women, 305 out of 961) and men (41.1% of all participating men, 1324 out of 3222).

Detailed results of ophthalmological history are published in Table S3.

Ophthalmological Examination

We report the available size (n) of the study group (n = 4183) when presenting results on these variables.

Visual acuity (right n = 4177, left n = 4173) and objective refraction (right n = 4169, left n = 4167). Two subjects had missing data for visual acuity for both eyes and 12 subjects had a result for visual acuity only for one eye. 4147 out of 4183 subjects (99.1%) had a visual acuity ≥0.5 in the better eye and 3665 out of 4183 (87.6%) subjects had a visual acuity ≥0.8 in the better eye (Table 7).

Considering the two time periods of the last visit to an ophthalmologist, there was no significant difference in visual acuity (Fisher's exact test: $p = 0.289$ for visual acuity ≥0.5 and Fisher's exact test: $p = 0.221$ for visual acuity ≥0.8) (Table 8).

The mean spherical equivalent for right eyes was −0.72 dpt±2.17 dpt (range: −15.25 dpt to 9.63 dpt) and for left eyes −0.72 dpt±2.20 (range: −17.13 dpt to 12.25 dpt).

Central Corneal Thickness (CCT) (right n = 4181, left n = 4181). Mean CCT was 538.2 μm±32.7 μm (min 375 μm, max 657 μm) for right eyes and 538.7 μm±33.0 μm (range 370 μm to 653 μm) for left eyes.

Intraocular Pressure (IOP) (right n = 4182, left n = 4180). Mean IOP for right eyes was 16.0±3.3 mmHg and for left eyes 16.1±3.4 mmHg (from 2 to 41 mmHg for right eyes and from 8 to 42 mmHg for left eyes). 358 subjects (8.6%) had an IOP >21 mmHg in at least one eye and 177 (4.2%) had an IOP > 21 mmHg in both eyes. 30 (including 9 subjects with an IOP higher 21 mmHg in at least one eye) out of 4183 subjects already received treatment for glaucoma. The mean IOP of these 30 participants was for right eyes: 18.0±3.8 mmHg (from 10 to 26 mmHg) and for left eyes: 19.6±3.9 mmHg (from 12 to 28 mmHg).

Visual field (right n = 4121, left n = 3978). A reliable FDT perimetry could be performed for 3932 subjects for both eyes and for 235 subjects only for one eye (thereof 220 subjects with a unreliable FDT (FE >1/3 and/or FPE >1/3) in one eye and 15

Table 5. Distribution of gender and race for all participants (n = 4183).

	40–44 years	45–49 years	50–54 years	55–59 years	≥60 years	All together
**Gender**						
Male	872	1004	832	460	54	3222
Female	329	283	202	135	12	961
Together	1201	1287	1034	595	66	4183
**Race**						
Caucasian	1191	1275	1019	586	64	4135
Black	2	1	1	1	0	5
Other	1	6	8	5	1	21
Missing data	7	5	6	3	1	22

doi:10.1371/journal.pone.0098538.t005

Table 6. Distribution of gender subdivided in production sites and sites of administrative function (n = 4183).

	Specifics of each location				Total	
	Production sites		Sites of administrative function			
**Male**	**2930**	78.7%	**292**	63.5%	**3222**	77.0%
	90.9%		9.1%		100%	
**Female**	**793**	21.3%	**168**	36.5%	**961**	23.0%
	82.5%		17.5%		100%	
**Total**	**3723**	100%	**460**	100%	**4183**	100%

doi:10.1371/journal.pone.0098538.t006

subjects with no FDT available for at least one eye). 16 subjects had missing or unreliable FDT for both eyes.

Confocal scanning laser ophthalmoscopy (right n = 4105, left n = 4119). Good quality HRT images (standard deviation < 30 μm and good illumination) were obtained in 4063 (97.1%) of 4183 subjects for both eyes.

Fundus photography (right n = 4182, left n = 4179). Fundus photography could be performed for 4179 participants in both eyes and for 3 participants only in the right eye.

Due to abnormalities in fundus photography, perimetry and/or IOP, as well as low visual acuity <0.8 in one eye, 1882 (45.0%) of all participants received a recommendation to consult a general ophthalmologist (25.6% (1070) to objectify abnormalities in fundus photography, perimetry and/or IOP and 19.4% (812) to check refraction). Due to low visual acuity (<0.8 in one eye) 463 out of the above mentioned 1070 participants had to be checked additionally for refraction (Table 9).

Furthermore 39 out of 1070 participants showed abnormalities of retina/macula, 243 of optic disc, 83 of visual field. Depending on central corneal thickness 653 participants had an ocular pressure, which seemed to be individual too high. 32 participants had a reduced quality of fundus photography. Further 78 participants had a reduced visual acuity, which was not explained by objective refraction or any other pathological findings (Table 10).

Discussion

To our knowledge the Evonik-Mainz-Eye-Care-Study (EMECS) is the first large study of its kind, which screened for major eye disease in an occupational health care setting by augmenting occupational medicine examinations by ophthalmological procedures such as measuring intraocular pressure, pachymetry, confocal scanning laser ophthalmoscopy and fundus photography.

Similar approaches for screening for glaucoma or driving ability among workers were reported [28,29]. But Adler et al only performed fundus photography and a medical history among a working population [28] and Wagner tested only visual acuity, stereopsis, phoria, color vision and intraocular pressure, the latter in only 35% of the study group [29]. Both studies did not obtain the necessary data to diagnose glaucoma according to current definitions.

More related to our study is the pilot study by Meyer et al, in which they tried to figure out, if screening for glaucoma could be a useful supplement for daily occupational medicine examination [27]. However, for this screening they used only a non-contact tonometer and a nerve fiber analyzer in 392 participants. Visual fields and fundus photography were not available.

Screening programs in the last 30 years were evaluated for cost-effectiveness. Gottlieb et al [35] performed a detailed analysis on the value of glaucoma screening and developed a model allowing them to project the cost of screening approximately 1 million people of different age groups. It was found, that costs were always higher than benefits in particular for persons older than 59 years of age. Le Blanc [36] discussed the results and concluded that combinations of other and more modern technics may increase the effectiveness. Hernandes et al [37] reviewed systematically studies published until 1997 and concluded that there is insufficient economic evidence on which to base recommendations regarding screening. Vaahtonranta-Lehtonen et al [38] simulated an organized screening program in a population aged 50–79 years. The estimated costs of screening a population of 1 million people were calculated to amount to 30 million Euros whereas 3360 quality-adjusted life-years (QUALYs) were gained. The authors allocated 9023 Euros to each QUALY. In addition they estimated that 930 years of visual disability could be avoided for 701 persons. Peto and Tadros [7] reported on diabetic eye screening programs in the UK and concluded that screening for diabetic retinopathy has been shown to be cost-effective in health-economic terms. Schmier et al [39] performed a literature review on effectiveness studies and concluded that differences in methods have created barriers to understanding costs and benefits. In summary, cost-effectiveness of ophthalmologic screening programs is uncertain and further studies are necessary to identify conditions that help to organise screening programs as effective as possible. We believe that our approach combining modern ophthalmologic procedures with standard occupational health examinations may be an opportunity to approach effectiveness. In particular it has to be taken in account that the population screened is younger than 65 years of age which may help to increase the value of the program [35]. Moreover the specific situation of a screened working population should be considered. The development of eye diseases may cause the loss of the working place leading to severe social and economic losses. We will try to evaluate the effectiveness of our screening program in future analyses focusing on glaucoma.

In this study we were successful in recruiting 4183 employees of Evonik Industries between 40 and 65 years of age within the predetermined timeframe of 10 months. This corresponded to 32.1% of the screening population group.

The participation rate in our study varied from 17.9% to 60.5% at the different sites (Table 2). At four sites (site no. 2, 6, 12, 13) the demand for examination was higher than the available slots for examinations. At these sites all employees until a certain date of registration were included ("first come, first served"). We believe that advertising with glaucoma as an "eye catcher" and the "first come, first served" strategy may have influenced the propensity to take part in the screening. This is certainly reflected by the

Table 7. Distribution of visual acuity of the better eye (n = 4183).

age	visual acuity of the better eye							
	**1.0**	**0.8**	**0.63**	**0.5**	**0.32**	**0.2**	**0.1**	**missing data for both eyes**
**40–44 years** (n = 1201)	893	222	62	19	4	1	0	0
**45–49 years** (n = 1287)	846	267	134	28	7	2	2	1
**50–54 years** (n = 1034)	623	271	105	27	7	1	0	0
**55–59 years** (n = 595)	324	171	74	18	4	2	2	0
**≥60 years** (n = 66)	25	23	13	2	1	1	0	1
**All together** (n = 4183)	2711	954	388	94	23	7	4	2

doi:10.1371/journal.pone.0098538.t007

Table 8. Visual acuity dependent on the last visit to ophthalmologist.

Last visit to ophthalmologist		Visual acuity of the better eye					Total
		**not sufficient (<0.5)**	**not sufficient (<0.8)**	**Sufficient (≥0.5)**	**sufficient (≥0.8)**	**missing data for both eyes**	
**Missing data**	**n**	1	4	40	37	0	41
**≤3 Years**	**n**	22	319	2489	2192	2	2513
**>3 Years**	**n**	11	193	1618	1436	0	1629
**Total**	**n**	34	516	4147	3665	2	4183

cross-classified table: time since last visit to ophthalmologist versus visual acuity of the better eye. Fisher's exact test: $p = 0.289$ for visual acuity ≥0.5.
Fisher's exact test: $p = 0.221$ for visual acuity ≥0.8.
doi:10.1371/journal.pone.0098538.t008

Table 9. Recommendation to consult a general ophthalmologist for all participants subdivided in production sites and sites of administrative function (n = 4183).

Recommendation to consult a general ophthalmologist	Production sites		Site of administrative function		All together	
**None**	1989	53.4%	312	67.8%	2301	55.0%
**Due to abnormalities in fundus photography, perimetry, and/or intraocular pressure***	993	26.7%	77	16.7%	1070**	25.6%
**To check refraction, because of low visual acuity<0.8 in one eye**	741	19.9%	71	15.4%	812	19.4%
**All together**	3723	100%	460	100%	4183	100%

*for more detailed information see Table 10.
**due to low visual acuity (<0.8 in one eye) 463 out of 1070 participants had to be checked additionally for refraction.
doi:10.1371/journal.pone.0098538.t009

pronounced prevalence of history of glaucoma (0.9%) and of known ocular hypertension (0.3%).

Compared to the mean participation rate (32.1%) a reduced participation (23.1%) was caused at site no. 6, the largest location, by a predefined timeslot of only 8 weeks. The reason for predefined timeslots was the demand of the Department of Occupational Health of Evonik Industries to offer the project in an adequate way at every site with at least 1.5 weeks each (Table 2). At site no. 8 a reduced participation rate (17.9%) could be explained by local IT-problems, which led to a delayed start of advertising campaign of the project at this site. Site no. 12 and 13 decided to take part of the project at the end of the planning phase – with only 3 weeks remaining. This was causative for the reduced participation rates of 19.8% and 18.5%, respectively, at these sites. Taking these limitations into account, recruitment far higher than 32.1% would have been possible and underlines the high acceptance by the screening population group. This estimate is supported by the participation rate in the BASF Study II [29]. In this study 15 different teams were able to recruit 12373 out of 18910 (65.4%) employees of BASF (a further German chemical company) in a similarly aged population to our study (>40 years) within 2 months.

Furthermore Adler et al. was able to recruit 9602 employees (> 40 years of age) within 18 months [28]. However, the percentage share is unknown because the size of the whole study group is not mentioned.

We were able to conduct a prompt and uncomplicated off-site evaluation of all data of each participant. Every participant received a letter reporting results and noticeable findings within less than two weeks.

Mean age of the screening population was 48.4 (±5.4) years and 98.4% of all subjects were in the 40 to 60 year age range. This reflects the age distribution of the company, which is influenced by an early retirement program (possible retirement at 57 years, usually at an age of 65 years). No relevant difference was found when we compared the age distributions between participants and non-participants.

Thus, we examined a relatively young age group. Glaucoma is rare before the age of 40 years. Age related macular degeneration is defined to occur only after the age of 50 years. Diabetic retinopathy can occur at any age. Thus, we examined an age group in which prevalences and incidences for these major eye diseases may still be relatively low. On the other hand, detection of

Table 10. Recommendation to consult a general ophthalmologist due to abnormalities in fundus photography, perimetry and/or intraocular pressure (n = 1070).

Reason	Number of participants*
Abnormalities of retina/macula	39
Abnormalities of optic disc	243
Abnormalities of visual field	83
Abnormalities of individual ocular pressure (depending on central corneal thickness)	653
Reduced quality of fundus photography	32
Other reasons	9
Reduced visual acuity, which is not explained by objective refraction or any other pathological findings	78

*(multiple reasons possible).
doi:10.1371/journal.pone.0098538.t010

these diseases would offer the chance for early diagnosis and possible treatment and preservation of visual function.

961 of 4183 participants (23.0%) were female and 3222 of 4183 participants (77.0%) were male subjects (Table 5). Depending on the structural conditions in the chemical industry with around 27.6% female employees in Germany [40], such an unequal distribution of gender compared to other population based studies was not surprising. However, the ratio of participation rates (female to male: 1 to 3.5) differed from the gender distribution in this company, which was 1 to 5.2 in the age group of 40 to 65 years. The difference in the gender distribution between participants and non-participants was significant. Possible explanation for the difference in the gender distribution of participants and non-participants could be an increased awareness for a preventive checkup in women. This is supported by a representative survey (1003 women and 952 men), which was published in 2010. In this survey women showed a higher interest in all kinds of preventive check-ups than men, among others in screening for glaucoma with a number of participants of 34.6% for women and 25.7% for men [41].

1629 out of 4183 participants (38.9%) could be screened, which had never been seen by an ophthalmologist or had not been seen within the last 3 years. This includes only 31.7% of all participating women but 41.1% of all participating men and underlines again the higher interest for preventive check-up in women [41]. If eye exams were regularly linked to occupational medicine examinations this underrepresentation of male participation might be corrected.

There were only few participants (n = 34, 0.8%) with an insufficient visual acuity <0.5 in the better eye. However, there were 516 participants (12.3%) with a suboptimal visual acuity < 0.8 in the better eye (Table 8). By taking the regular visual acuity tests into account, which are performed by the Department of Occupational Health of Evonik Industries every 2–3 years (Table 1), the number of participants, that failed visual acuity < 0.8 was quite high. However, there was no significant difference for visual acuity between the two time periods of the last visit to an ophthalmologist (Table 8).

In summary the screening concept of the Evonik-Mainz-Eye-Care-Study seems to offer a feasible concept for screening for major eye diseases within the setting of occupational health care in industry.

Conclusions

To our knowledge, EMECS is the first large study with such a screening concept using modern ophthalmological examination techniques within the infrastructure of the existing occupational health care system. This article describes the study protocol, design and basic epidemiological characteristics of the screened population and will serve as a reference for future analyses, mainly on glaucoma, diabetic retinopathy and age-related macula degeneration.

We were successful in recruiting and examining more than 30% of the target working population. This investigation documents a successful cooperation between occupational health care in industry and an ophthalmological research department and could proof, that the screening concept of EMECS is a feasible method.

Supporting Information

Table S1 General medical history of all participants (n = 4183). (DOCX)

Table S2 General examination of all participants (n = 4183). (DOCX)

Table S3 Ophthalmological history of all participants (n = 4183). (DOCX)

Acknowledgments

The authors wish to thank the workers of Evonik Industries who attended the study and Prof. Thomas Erren, Institute and Policlinic for Occupational Medicine, Environmental Medicine and Prevention Research of Cologne University, and his staff for reviewing an earlier version of the manuscript. We thank two unknown reviewers for their helpful comments on an earlier version of the manuscript.

Author Contributions

Conceived and designed the experiments: LB JW SH RB UM NP. Performed the experiments: LB CD AL. Analyzed the data: LB JW PM SH. Wrote the paper: LB JW PM SH NP.

References

1. Resnikoff S, Pascolini D, Etya'ale D, Kocur I, Pararajasegaram R, et al. (2004) Global data on visual impairment in the year 2002. Bull World Health Organ 82: 844–851.
2. Lim LS, Mitchell P, Seddon JM, Holz FG, Wong TY (2012) Age-related macular degeneration. Lancet 379: 1728–1738.
3. Sivaprasad S, Gupta B, Crosby-Nwaobi R, Evans J (2012) Prevalence of diabetic retinopathy in various ethnic groups: a worldwide perspective. Surv Ophthalmol 57: 347–370.
4. Buch H, Vinding T, Nielsen NV (2001) Prevalence and causes of visual impairment according to World Health Organization and United States criteria in an aged, urban Scandinavian population: the Copenhagen City Eye Study. Ophthalmology 108: 2347–2357.
5. de Voogd S, Ikram MK, Wolfs RC, Jansonius NM, Hofman A, et al. (2005) Incidence of open-angle glaucoma in a general elderly population: the Rotterdam Study. Ophthalmology 112: 1487–1493.
6. Quigley HA, Broman AT (2006) The number of people with glaucoma worldwide in 2010 and 2020. Br J Ophthalmol 90: 262–267.
7. Peto T, Tadros C (2012) Screening for diabetic retinopathy and diabetic macular edema in the United kingdom. Curr Diab Rep 12: 338–345.
8. Tuomi K, Huuhtanen P, Nykyri E, Ilmarinen J (2001) Promotion of work ability, the quality of work and retirement. Occup Med (Lond) 51: 318–324.
9. Costa G, Sartori S (2007) Ageing, working hours and work ability. Ergonomics 50: 1914–1930.
10. Sharit J, Czaja SJ (1994) Ageing, computer-based task performance, and stress: issues and challenges. Ergonomics 37: 559–577.
11. Sundling V, Gulbrandsen P, Straand J (2013) Sensitivity and specificity of Norwegian optometrists' evaluation of diabetic retinopathy in single-field retinal images - a cross-sectional experimental study. BMC Health Serv Res 13: 17.
12. Mowatt G, Burr JM, Cook JA, Siddiqui MA, Ramsay C, et al. (2008) Screening tests for detecting open-angle glaucoma: systematic review and meta-analysis. Invest Ophthalmol Vis Sci 49: 5373–5385.
13. Burr JM, Mowatt G, Hernandez R, Siddiqui MA, Cook J, et al. (2007) The clinical effectiveness and cost-effectiveness of screening for open angle glaucoma: a systematic review and economic evaluation. Health Technol Assess 11: 1–190.
14. Antony K, Genser D, Froschl B (2007) Validity and cost-effectiveness of methods for screening of primary open angle glaucoma. GMS Health Technol Assess 3: 1–113.
15. Boivin JF, McGregor M, Archer C (1996) Cost effectiveness of screening for primary open angle glaucoma. J Med Screen 3: 154–163.
16. Yamauchi Y, Kemma H, Goto H, Nakamura A, Nagaoka T, et al. (2012) Novel automated screening of age-related macular degeneration. Jpn J Ophthalmol 56: 577–583.
17. Hernandez RA, Burr JM, Vale LD (2008) Economic evaluation of screening for open-angle glaucoma. Int J Technol Assess Health Care 24: 203–211.
18. Antony K, Genser D, Froschl B (2007) Validity and cost-effectiveness of methods for screening of primary open angle glaucoma. GMS Health Technol Assess 3: 57.
19. DGUV (2010) Deutsche Gesetzliche Unfallversicherung: Berufsgenossenschaftliche Grundsätze für arbeitsmedizinische Vorsorgeuntersuchungen. Stuttgart: Gentner.

20. HSE editor (2000) Health and Safety Guidance 6 (HSG6): Safety in working with lift trucks: HSE Books.
21. HSE (2003) Work with display screen equipment: Health and Safety (Display Screen Equipment) Regulations 1992 as amended by the Health and Safety (Miscellaneous Amendments) Regulations 2002: HSE Books. 76 p.
22. OSHA (1989) Occupational Safety and Health Administration: Pre-Employment Medical Examination Program, http://www.osha.gov/pls/oshaweb/owadisp.show_document?p_table = DIRECTIVES&p_id = 1696.Accessed 2014 May 11.
23. OSHA (1989) Occupational Safety and Health Administration: Medical Examination Program for Compliance Safety and Health Officers, http://www.osha.gov/pls/oshaweb/owadisp.show_document?p_table = DIRECTIVES&p_id = 1697.Accessed 2014 May 11.
24. DOT The U.S. Department of Transportation: Visual Requirements for Commercial Motor Vehicle Drivers, http://www.fmcsa.dot.gov/facts-research/research-technology/tech/TB-01-007.htm.Accessed 2012 Dec 3.
25. FAA (2006) Federal Aviation Administration: Guide for Aviation Medical Examiners - Synopsis of Medical Standards, http://www.faa.gov/about/office_org/headquarters_offices/avs/offices/aam/ame/guide/standards.Accessed 2014 May 11.
26. DGUV (2007) Deutsche Gesetzliche Unfallversicherung : Guidelines for Occupational Medical Examinations - Prophylaxis in Occupational Medicine. Stuttgart: Gentner.
27. Meyer C (2006) Ist ein Glaukomscreening in Großunternehmen eine sinnvolle arbeitsmedizinische Ergänzung der üblichen Vorsorgeuntersuchung? http://edisssubuni-hamburgde/volltexte/2007/3323/pdf/Christine_Meyerpdf Accessed 2014 May 11.
28. Adler W, Warntges S, Lausen B, Michelson G (2010) [Prevalence of glaucomatous optic nerve atrophy among a working population in Germany diagnosed by a telemedical approach]. Klin Monbl Augenheilkd 227: 905–911.
29. Wagner G (1974) Sehvermögen - Farbtüchtigkeit - Augeninnendruck. - Methodik und Ergebnisse einer Vorsorgeuntersuchung in einem chemischen Großbetrieb. - BASF-Studie II: F.K. Schattauer Verlag Stuttgart-New York. 97 p.
30. DOG (2013) Fahreignungsbegutachtung für den Straßenverkehr. Empfehlungen der Deutschen Ophthalmologischen Geseleeschaft (DOG) und de Berufsverbandes der Augenärzte Deutschlands (BVA). http://www.dog.org/wp-content/uploads/2014/03/DOG_Fahreignungsbegutachtung_2014_03.pdf. Accessed 2014 May 11.
31. EC (1991) European Community: Official Gazette No. L 2371/20.
32. (1991) Grading diabetic retinopathy from stereoscopic color fundus photographs–an extension of the modified Airlie House classification. ETDRS report number 10. Early Treatment Diabetic Retinopathy Study Research Group. Ophthalmology 98: 786–806.
33. Rothman KJ, Greenland S, Lash TL (2008) Modern Epidemiology. Philadelphia: Lippincott Williams & Wilkins.
34. StataCorp (2009) Stata: Release 11. Statistical Software. College Station. Texas: StataCorp LP.
35. Gottlieb LK, Schwartz B, Pauker SG (1983) Glaucoma screening. A cost-effectiveness analysis. Surv Ophthalmol 28: 206–226.
36. Le Blanc R (1989) Value of intraocular pressure measurement as a screening tool for glaucoma. Surv Ophthalmol 33 Suppl: 445–446; discussion 449–450.
37. Hernandez R, Rabindranath K, Fraser C, Vale L, Blanco AA, et al. (2008) Screening for open angle glaucoma: systematic review of cost-effectiveness studies. J Glaucoma 17: 159–168.
38. Vaahtoranta-Lehtonen H, Tuulonen A, Aronen P, Sintonen H, Suoranta L, et al. (2007) Cost effectiveness and cost utility of an organized screening programme for glaucoma. Acta Ophthalmol Scand 85: 508–518.
39. Schmier JK, Halpern MT, Jones ML (2007) The economic implications of glaucoma: a literature review. Pharmacoeconomics 25: 287–308.
40. BAVC (2002) Informationsbrief für Führungskräfte Bundesarbeitgeberverband Chemie eV 10/2002.
41. GFK-Marktforschung (2010) Hitliste der Vorsorgeuntersuchungen. Apotheken Umschau 12/2010.
